# The interaction of orthography, phonology and semantics in the process of second language learners’ Chinese character production

**DOI:** 10.3389/fpsyg.2023.1076810

**Published:** 2023-03-02

**Authors:** Linlin Zhang, Hongbing Xing

**Affiliations:** Institute on Educational Policy and Evaluation of International Students, Beijing Language and Culture University, Beijing, China

**Keywords:** Chinese character production, connectionism, orthography, phonology, semantics

## Abstract

Theories of connectionism emphasize the mappings of orthography, phonology, and semantics in the process of word recognition and production. Chinese has a logographic writing system, which is markedly different from alphabetic languages. The current study investigated how orthography, phonology, and semantics contribute to Chinese character production among Chinese as a second language (CSL) learners. This study collected 33,856 Chinese characters in a sample of 2,116 CSL learners with 7 diverse L1s. ANOVA was conducted to examine the effect of Chinese character error type on 7 L1s and three Chinese proficiency levels. The results of ANOVA revealed that successful CSL learners’ Chinese character production was derived from connections between orthography, semantics, and phonology. Semantics, especially the orthography-semantics connection, was the key point for production skills. Furthermore, connectionist models of languages rather than language distance affected production. These findings indicate that Chinese character production is associated with not only orthographic knowledge but also representation mechanisms of orthography, phonology, and semantics between different language writing systems. The results contribute to a better understanding of literacy skills in CSL learners. Future research could further address how CSL learners transform perceptive skills into production skills and the correlation between reading and writing skills by examining and controlling other important cognitive variables.

## Introduction

Literacy skills consist of the two components of reading and writing, and both are held to be necessary for successful language acquisition. Chinese, which is written with an inventory of thousands of Chinese characters (or *hanzi*), is usually considered a logography, a system in which the basic writing units correspond to units of meaning (i.e., morphemes; Perfetti and Tan, 1998; Tan et al., 2001; Sung and Wu, 2011; Kuo et al., 2015). This is largely different from alphabetic languages where a smaller inventory of 20–40 semantically devoid symbols (i.e., letters) correspond to sounds and word recognition is driven by grapheme-to-phoneme conversion rules (Shen, 2005; Loh et al., 2018; Li et al., 2021). Chinese characters are themselves composed in a semi-regular fashion of components that often suggest semantic and/or phonetic characteristics of the morpheme. However, it is never the case in Chinese that a phonetic component maps onto a subsyllabic phonological representation in the way that a letter maps onto a substring of a word’s phonological form in an alphabetic system (Perfetti et al., 2005). The Chinese character system presents an interesting conundrum to traditional letter-processing-based models of word recognition and production, especially for Chinese as a second language (CSL) learners (Williams and Bever, 2010; Loh et al., 2018; Chai and Ma, 2022).

While many studies have shown that writing is strongly correlated with reading acquisition among native Chinese learners (Pak et al., 2005; Chan et al., 2006; Zhang and Reilly, 2015) and CSL learners (Guan et al., 2011; Xu et al., 2013), they are two different cognitive activities. Reading involves accessing meaning by decoding printed words, while writing involves producing printed words *via* phonological and semantic representation (Zhang and Roberts, 2021a,b). Contradictory findings on the writing-on-reading effect have been reported (Bi et al., 2009; Zhai and Fischer-Baum, 2019). As argued elsewhere (Ke, 1996; Ke, 1998), successful recognition of a character could occur when enough graphic details of a character match those of a character existing in memory; for character production, however, one must have complete knowledge of the character and then transform that knowledge into motor skills. Briefly, partial information can lead to recognition, but total mastery of the character is required for accurate production. Chinese character production involves not only central processes underlying conceptual preparation, lexical selection, and orthographic access but also peripheral processes of motor programming and actual writing execution (Baxter and Warrington, 1986; Van Galen, 1991; Wang et al., 2020). Frequency-related factors (Kandel and Perret, 2015; Qu et al., 2016), semantic variables (Coltheart et al., 1988), and phonological variables (Afonso and Álvarez, 2011) have been shown to be important factors in transcoding semantic information into orthographic output. However, compared with perceptive skills, production skills have been somewhat ignored by CSL researchers (Zhang, 2014). While some research has been carried out on the relationship between Chinese character recognition and production (Ke, 1996), on how learners process Chinese orthographic units (Everson and Ke, 1997), on learning strategies (Sung and Wu, 2011), on stroke orders (Zhang, 2014; Chang et al., 2015), on psycholinguistic variables (Wang et al., 2019), on phonological awareness (Zhang and Roberts, 2021a,b), and on mechanisms underlying production (Guan et al., 2021), the analyses do not specifically address the issue of how the three variables of orthography, phonology and semantics connect in the production process and whether CSL learners’ language backgrounds and Chinese proficiency levels affect production. Thus, this study addresses the effects of orthographic, phonological, and semantic processing in the production of Chinese characters by CSL learners through big data from a national CSL proficiency test, Hanyu Shuiping Kaoshi (HSK), to further explore whether the productive errors that learners make are semantically oriented or phonologically oriented and what characteristics of the developmental errors learners make producing Chinese characters. The findings of the present study will add to our understanding of the connectionist model of character production, which can provide the foundation to examine the brain mechanisms underlying the acquisition of writing abilities in CSL learners.

### Literature review

The literature review consists of three sections. The first section focuses on connections of orthography, phonology and semantics of Chinese characters, the second section deals with orthographic, phonological and semantic knowledge in Chinese children and the third section deals with the knowledge of orthography, phonology and semantics among CSL learners. The research questions were put forward after the literature review.

### Connections of orthography, phonology and semantics of Chinese characters

The mental lexicon model of Chinese characters consists of connections of orthography, phonology, and semantics, among which connection strength and connection direction play important roles during cognitive processing (Li, 2002; Chang et al., 2015; McBride, 2016). The representation of characters has two bidirectional pathways, the first being directly connected from orthography to phonology and semantics, and the other being indirect access through components, phonetic radicals, and semantic radicals (Chen and Peng, 1994; Ventura et al., 2007). Moreover, orthographic, phonological, and semantic information at both radical and character levels could be activated simultaneously (Zhou and Marslen-Wilson, 1999). Thus, a complex sublexical system of Chinese characters is established, forming multiple connection networks. Xing (2016) constructed a neural network model of Chinese compound characters *via* distributed representation analysis, finding that there was a symbolic distribution of orthography-phonology and orthography-semantics connections in the brain (Dayhoff, 1991; Fausett, 1994; Anderson, 1995).

### Orthography-phonology connection of Chinese characters

In logographic writing systems such as Chinese, the orthography-phonology connection is bidirectional, and the activation can be from phonology to orthography, or vice versa (Song et al., 1995; Shu et al., 2008). However, the connection strength of the two directions is not entirely equal. Taking the character “礴” (pronounced “bo2,” majestic) (all numbers indicate tones) as an example, the connection from orthography to phonology is strong and easy to extract, while access to orthography from phonology is difficult to activate. In addition to the analysis of connection strength, connection frequency has been used to further explore the correspondence between orthography and phonology (Worlton, 2014; Li et al., 2018). In Chinese, not only is there a lot of homophony—several characters share a given pronunciation, but many Chinese characters can be read with a variable number of pronunciations. This means that the orthography-phonology connection can be multinodal (Shu and Zhang, 1987; Van Orden and Goldinger, 1994; Shu et al., 1998). In other words, the same syllable including tone can be represented by distinct characters and have completely different meanings (Kuo et al., 2015). For example, the syllable “ba1” has a multinodal connection with characters such as “八,” “巴,” “芭,” and “粑” (all pronounced “ba1”), while the character “行” is only connected with two syllables (pronounced “xing2,” capable and “hang2,” line) (Xing, 2020). While the orthography-phonology connection is less reliable in the Chinese writing system, phonetic radical awareness, the knowledge of and the ability to manipulate the functional and positional information of phonetic radicals play an important part in the development of Chinese literacy (Zhang and Roberts, 2019).

Although there is no direct mapping between phonology and orthography in Chinese characters, the orthography-phonology connection can be described from the perspective of phonetic radicals (Shu et al., 1993; Zhang and Roberts, 2019). The phonetic radicals indicate the pronunciation of a character, and the reliability with which orthography corresponds with its phonology may be described in two ways: regularity and consistency (Fang et al., 1986; Hue, 1992; Shu et al., 2000a). Regularity denotes the degree to which the pronunciation of a character matches that of its phonetic radical (Lin and Collins, 2012). Consistency is defined as the degree to which a set of characters with the same phonetic radical share the same pronunciation, regardless of whether the phonetic radical’s pronunciation is the same or differs from that of the character (Fang et al., 1986). Like regularity, the consistency of Chinese characters has been found to influence speed and accuracy in character naming (Lee et al., 2004; Shu and Wu, 2006). Some eye tracking studies showed facilitation of phonological access for consistent over inconsistent Chinese characters (Tsai et al., 2004; Pan et al., 2021). While the orthography-phonology connection is less reliable in the Chinese writing system, phonetic radical awareness, the knowledge of and the ability to manipulate the functional and positional information of phonetic radicals play an important part in the development of Chinese literacy (Zhang and Roberts, 2019).

### Orthography-semantics connection of Chinese characters

Chinese characters are not formed by letters as in alphabetic languages; rather, they represent morphemes, which in turn form words (Shen and Ke, 2007). Morphemes are flexible in use and complex in systems (Nagy et al., 2002; Huang and Liao, 2017). Thus, the orthography-semantics connection may be even more complex than in English, and it can be described from the standpoint of polysemy, synonymy and semantic radicals. The first, polysemy, means a word carrying two or more meanings, forming multidimensional connections between orthography and semantics. For example, the word “深” (pronounced “shen1”) carries many meanings, such as large distance from top to bottom or from inside to outside, depth, profound, very, and long time from the beginning. Moreover, the connection strength is determined by frequency, forming the dynamic distribution relationship and frequency knowledge of polysemy. Synonymy implies that the same meaning can be manifested by different words. To illustrate, “家父” (pronounced “jia1 fu4”), “令尊” (pronounced “ling4 zun1”), “父亲” (pronounced “fu4 qin1”), “爸爸” (pronounced “ba4 ba”), “老爹” (pronounced “lao3 die1”) all refer to “father.”

Semantic radicals imply the meaning of a character. Characters sharing the same radicals are usually related in meaning and fall into the same semantic category (Tong et al., 2009; Kuo et al., 2015). According to whether a character is semantically transparent or opaque, the characters are divided into radical-transparent and radical-opaque characters. The meaning of a transparent character is directly related to the meaning of its semantic radical (Hsiao et al., 2007). For example, the characters “树” (pronounced “shu4,” tree), “林” (pronounced “lin2,” forest) share the same radical “木” (pronounced “mu4,” wood). In contrast, the meaning of an opaque character is not directly related to the meaning of its semantic radical. For example, the character “燕” (pronounced “yan4,” swallow) does not contain the radical “鸟” (pronounced “niao3,” bird) and is, therefore, an opaque character. The knowledge of semantic radicals and their orthographic functions plays an important role in the process of character recognition and production. Some studies provide strong evidence supporting this demonstration (Feldman and Siok, 1999; Taft and Chung, 1999; Yan et al., 2012; Wong, 2017). Shen (2000) investigated the role of the knowledge of radicals and its relation to both character recognition and character production among beginning and intermediate college learners of Chinese and found that semantic radicals served as processing units and learners’ radical knowledge affects character learning. Yan et al. (2012) reported a larger semantic priming effect from radical-transparent than opaque characters during sentence reading.

### Orthographic knowledge of Chinese characters

Chinese characters are constructed *via* three orthographic tiers: strokes, components and whole characters (Shen, 2005; Loh et al., 2018). Some studies have noted that stroke order (Zhang, 2014), the number of strokes (Just and Carpenter, 1987), and the number of stroke patterns (Chen and Liu, 2000) could affect Chinese character recognition and production. The components refer to the smallest stroke patterns that can be recursively used as a meaningful unit to form a character (Taft and Zhu, 1997; Loh et al., 2018). Shi (2021) demonstrated that the frequency knowledge of components, manifested as the connection knowledge between the attributes of components and composed characters, was the core of orthography. The combination rules of components make Chinese orthography more complicated because most components are placed in different positions across characters, and some components only reside in specific positions (Loh et al., 2018). To illustrate, the component “女” [female] can appear on both the left side (such as “娘” [mother], “好”[good]) and the right side (such as “妆” [make up], “汝”[you]), while the component “氵” [water] only resides on the left side (such as “海”[sea]), and the component “刂” [knife] only appear on the right side (such as “剑”[sword]). In addition, the components are different from the radicals that cue the meaning or pronunciation of characters. For instance, the character “烨” (pronounced “ye4,” bright) contains three components: “火” [fire], “化” [change] and “十” [ten], but only the component “火” is related to the meaning of the whole character and classified as a radical. The third tier, whole character, can be categorized into two types: simple and compound characters (e.g., “女” [woman] and “妈”[mother]) (Sung and Wu, 2011; Loh et al., 2018). The frequency knowledge of compound characters is more complex, as the frequency distribution of the component combination mode, component function and position regularity may all differ.

### Orthographic, phonological and semantic knowledge in Chinese children

Considerable research has been carried out on the recognition of Chinese characters, focusing on how orthographic, phonological, and semantic properties are activated to lead to visual recognition (Shen, 2005; Tong and Yip, 2015; Zhang H. et al., 2020). The dual route model to lexical recognition has been proposed to explain the visual presentation of a character, that is, the first being indirect through recognition of the word’s phonology, and the other being direct access between orthography and semantic category (Zhou and Marslen-Wilson, 1999; Williams and Bever, 2010). Phonological information mediates access to meaning in Chinese, and phonological activation occurs earlier than semantic activation during word reading (Tan and Perfetti, 1997; Perfetti and Tan, 1998). In contrast, there is much evidence suggesting that character meaning can be directly accessed from orthography (Chen et al., 1995; Zhou and Marslen-Wilson, 1999, 2000; Zhou et al., 1999; Chen and Shu, 2001; Zhang H. et al., 2020). For example, Zhou et al. (1999) revealed that phonology had no inherently privileged role over orthography in constraining semantic activation. Eye-tracking evidence was also in favor of the direct route, reporting that semantic information was available very early (Pan et al., 2020), and that semantic priming was earlier and larger than phonological priming in Chinese (Yan et al., 2009; Pan et al., 2022). Shu et al. (2005) argued that the dual route model failed to explain the oral reading of Chinese characters because oral reading in Chinese involved contact with lexical representations as well as sublexical units. At the same time, a different view of the triangle model has been developed by Seidenberg and McClelland (1989), who noted that reading involved concurrent orthographic, phonological and semantic processing, all linked *via* two bidirectional pathways. Given that the mappings between semantics and orthography are more systematic in Chinese than the mappings between orthography and phonology, the orthography-semantics connection is much more reliable. A study of automatic semantic influence on early visual word recognition reflected this unique process of Chinese reading (Wang et al., 2019).

Existing research studies have explored development of orthographic knowledge in Chinese native children. For instance, Loh et al. (2018) showed that Chinese children tended to notice the components at a very early age and researchers have paid more attention to position regularity awareness development (Chan and Nunes, 1998; Pak et al., 2005; Anderson et al., 2013). However, the knowledge of character structure has not yet been extensively studied in existing research (Yeh and Li, 2002). Phonological awareness and phonetic radical awareness are both important type of metalinguistic processing skills in character recognition for native Chinese speakers (Shu et al., 2000b), and the former is mediated by the latter (Ho and Bryant, 1997). While phonological awareness did not significantly predict the children’s performance in character writing (Zhang and Roberts, 2019), the contribution of phonetic radical awareness to character writing has been documented in a study by Yin and McBride (2015). Given the importance of phonology and semantics in Chinese Characters, more researchers have explored the relationship of phonology and semantics with other skills (Zhao et al., 2012; Ye et al., 2021). For instance, Mcbride-Chang et al. (2005) tested three different visual skills, along with Chinese character recognition, vocabulary, speeded naming, and syllable deletion skills twice over one school year among 118 Hong Kong and 96 Xiangtan, China kindergartners. The results suggested a bidirectional association of visual skills with Chinese character acquisition across scripts. Wang et al. (2018) explored how semantic radicals in Chinese characters facilitate hierarchical category-based induction and provided electrophysiological evidence that semantic radicals may improve sensitivity to distinguish between hierarchical concepts.

### Orthographic, phonological and semantic knowledge among CSL learners

There has also been increasing interest in conducting research on the acquisition of the Chinese orthographic system by CSL learners, focusing on four key themes: teaching and learning strategies, the teaching of recognition and production study, character knowledge and awareness, and computer-assisted language learning (Yeh et al., 2003; Shen, 2005; Allen, 2008; Sung and Wu, 2011; Ye, 2013; Ke, 1996; McBride, 2016 Li, 2020). Many studies have examined the development of CSL learners’ acquisition of orthographic knowledge (Shi, 1999; Shi, 2000; Loh et al., 2018), such as radical awareness (Shen and Ke, 2007; Kuo et al., 2015; Tong and Yip, 2015; Wong, 2017), phonological awareness (Zhang and Roberts, 2019, 2021a,b; Wang et al., 2020), position regularity (Wang et al., 2003, 2004), and stroke order (Zhang, 2014; Chang et al., 2015). For instance, Zhang and Roberts (2019) explored the different roles that phonological awareness and phonetic radical awareness played in the development of character literacy skills with English and Arabic CSL learners, finding that the learners’ phonological awareness, but not their phonetic radical awareness, predicted the acquisition of character reading and writing skills. Nonetheless, Chinese character acquisition is a process of learning the connections of orthography, phonology, and semantics, which relies more on the correspondence between orthography and semantics. Moreover, orthographic knowledge differs from linguistic properties (e.g., semantic or phonetic clues), whereas previous studies tended to mix the effects of the two, making it difficult to separate the role of orthographic knowledge from functional information (Tong and McBride, 2014; Loh et al., 2018).

Language backgrounds play important role in the processing of Chinese characters. There have been analyses of the individual characteristics of learners from different language backgrounds (Ke, 1998; Zhang, 2008; Li et al., 2014; Zhang, 2016; Zhang and Roberts, 2019). For instance, Sung and Wu (2011) investigated factors influencing the learning of Chinese characters and an interactive effect was found among gender, language background, and previous foreign language learning experiences on strategies of paying attention to the characters. Chai and Ma (2022) revealed the role of cultural background in the relationships between Chinese handwriting and reading comprehension. This may reflect the influence of first language orthographic experience on second language decoding and word learning (Jiang, 2003; Hamada and Koda, 2008; Lin and Collins, 2012; Tong et al., 2016; Zhang and Roberts, 2021a,b). However, whether Ll-L2 orthographic distance influences performance differences and facilitates L2 word recognition, as argued by Koda (1996), requires further research. In addition to the language background, developmental characteristics or Chinese proficiency levels of CSL learners also affect the acquisition of characters (Yeh et al., 2003; Li et al., 2014). For instance, Li et al. (2014) claimed that with the improvement of proficiency levels, the orthographic awareness of foreign students would be developed accordingly. This study explore whether the productive errors that learners make are semantically oriented or phonologically oriented and what characteristics of the developmental errors learners make producing Chinese characters. Error analysis was employed to analyze learners’ mistakes in second language learning and to help second language teachers develop theories of language teaching and learning to achieve effective instruction (Schumann and Stenson, 1975; Leki, 1991; Ellis, 1994; Xiao, 2002). For instance, Lee (1997) described an investigation into ESL students’ performance in error correction in writing and discussed the pedagogical implications which arise from the study. And it was found that students’ major difficulty in error correction lied in their failure to detect errors rather than the lack of knowledge. As these errors did not occur by chance but, rather, by reason (Corder, 1967), they were systematic and may represent either a transitional stage in the development of a grammatical rule or the final stage of the speakers’ knowledge. Thus, analysis of the error type was necessary to explore the productive errors in Chinese characters.

### The current study

The current study focuses on the effects of orthographic, phonological, and semantic processing in the production of Chinese characters by CSL learners. Specifically, the study explores whether language backgrounds and Chinese proficiency levels interact with productive errors of Chinese characters. The following research questions guide the present investigation:

1. Does orthography-semantics connection plays a more important role than orthography-phonology connection and orthographic knowledge in Chinese character production?
2. Is there any difference in the processing of orthography, phonology and semantics of Chinese characters by CSL learners with different language backgrounds?

## Materials and methods

### Participants

This study selected CSL learners from a large-scale database, containing information on examinees who participated in the HSK test at various locations in China in 2008, 2009, and 2010. A total of 2,271 participants who took part in seven sets of the HSK Elementary-Intermediate test were selected, 18 participants with blank answer cards and 137 Arabic participants were excluded due to the small number of participants. The participants came from 82 countries distributed in 10 language backgrounds (Japanese, Korean, Russian, English, Mongolian, Thai, German, Spanish, Arabic, French). German, French and Spanish were merged into European language due to the small number of participants. Thus, a total of 2,116 CSL learners with 7 language backgrounds were selected to participate in this study. The proficiency levels were identified on the report card after each examination. The study merged Elementary C and Elementary B Chinese proficiency levels to low level, Elementary A and Intermediate C Chinese proficiency levels to middle level, Intermediate B and Intermediate A to high level. The learners in each language background group were distributed over three different levels of Chinese proficiency: low level, middle level and high level.

### HSK Chinese character writing proficiency test (elementary-intermediate)

**Figure 1:**
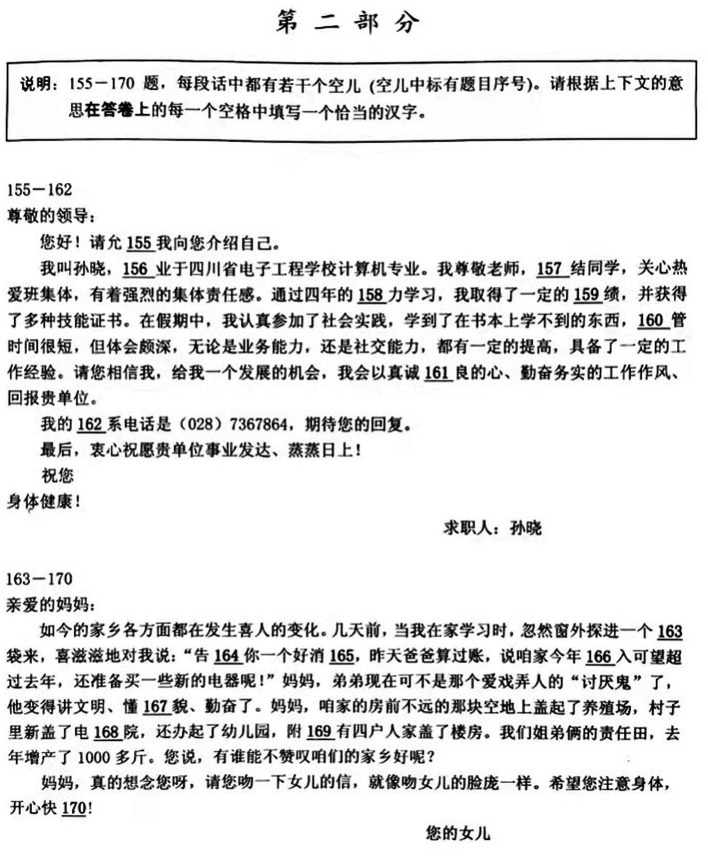
Sample test in the HSK character writing test.

The HSK (short for “Hànyuˇ Shui˘píng Kăoshì” and literally translating to Chinese Proficiency Test) is the most widespread standardized test for assessing the Chinese proficiency of nonnative speakers. The HSK (Elementary-Intermediate) consists of four subtests (i.e., listening [50 items], grammar structure [30 items], reading [50 items], and cloze [40 items]) for a total of 170 items. Of these, items 1–154 are multiple choices, and items 155–170 are fill-in-the-blanks (Chai and Ma, 2022). This study focused on the results of the character writing part of the cloze section. This part mainly examined students’ orthographic competence and their mastery of lexical words and writing in Chinese as an L2. Test-takers filled in 16 blanks (one point per blank) in the orthography section with appropriate and correct handwritten Chinese characters based on three to four supplied passages. The cloze section lasted for 30 min (Chai and Ma, 2022). The sample test in the second part of the HSK reading test is shown in Figure 1.

### Materials

Each set of HSK (Elementary-Intermediate) tests contained 16 Chinese characters, and there were 112 Chinese characters in seven sets of HSK tests (no characters were repeated across the selected test sessions). A total of 2,116 valid samples were selected, with 16 Chinese characters written by each subject, for a total of 33,856 Chinese character samples. The target characters of the seven sets of HSK tests and the distribution of sampling data are shown in appendix (see Supplementary Appendix Tables 1, 2).

### Annotation specification of productive characters

The patterns of mistakes that learners made on character production were classified in three ways: orthographic errors, orthography-phonology connection errors and orthography-semantics connection errors. The annotation specification of Chinese characters is discussed as follows.

### Orthographic errors

The orthographic errors were divided into three types: structural errors, component errors, and stroke errors.

1. Structural errors refer to errors in the structure and position of Chinese characters. For example, the target character is “静” (pronounced “jing4,” quiet), but the produced character is 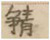.
2. Component errors refer to component ambiguity or translocation. For example, the target character is “努” (pronounced “nu3,” put forth strength), but the produced character is 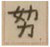.
3. Stroke errors refer to a defective stroke, a change in stroke shape, or an extra stroke. For example, the target character is “乐” (pronounced “le4,” happy), but the produced character is 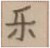.

### Orthography-phonology connection errors

There were two types of orthography-phonology connection errors: homophone substitution and sublexical errors of phonetic radicals.

1. Homophone substitution refers to replacing the target character with homophonic or near pronunciation Chinese characters. For example, the target character is “班” (pronounced “ban1,” class), but the produced homophone is “般” (pronounced “ban1,” like).
2. Sublexical errors of phonetic radicals refer to the substitution of target characters by the same or near phonetic radicals. For example, the target character is “境” (pronounced “jing4,” boundary), but the produced character is “竟” (pronounced “jing4,” unexpectedly).

### Orthography-semantics connection errors

The errors in the orthography-semantics connection of Chinese characters can be divided into two types: analogy and sublexical errors of semantic radicals.

1. Analogy refers to the substitution of target characters by Chinese characters with similar or related meanings. Characters with similar meanings, as when the target character is “舒” (pronounced “shu1,” comfortable), but the produced character is “服” (pronounced “fu2,” be accustomed to). Characters with related meanings, as when the original meaning of the word is “批准” (pronounced “pi1 zhun3,” approval), the target character is “批” (pronounced “pi1,” approval), but the produced character is “标” (pronounced “biao1,” mark).
2. Sublexical errors of semantic radicals refer to replacing target characters with characters of the same or a similar semantic radical. For instance, the target character is “谈” (pronounced “tan2,” talk), but the produced character is “讲” (pronounced “jiang3,” speak).

### Nonstandard characters

Nonstandard characters were divided into two types: non-Chinese characters and blank characters.

1. Non-Chinese characters refer to those written in Chinese pinyin or symbols that cannot be identified as Chinese characters.
2. Blank means no answer.

### Other Chinese characters

Other Chinese characters were divided into irrelevant Chinese characters and traditional Chinese characters.

1. Irrelevant Chinese characters refer to the output of a Chinese character that has correct orthography, semantics, and phonology, but it is not the target character.
2. Traditional Chinese characters mean that Chinese characters or components are all traditional ones.

### Other types of errors

Errors other than those mentioned above.

### Data analysis

Three types of errors (orthographic errors, orthography-phonology connection errors, and orthography-semantics connection errors) made by CSL learners were first calculated to examine the error rates. Then, this study conducted 3 (Chinese character error types: orthographic errors, orthography-phonology connection errors, and orthography-semantics connection errors as within-subject factors) × 3 (Chinese proficiency levels: low level, middle level and high level as between-subject factors) × 7 (Language backgrounds: Japanese, Korean, Russian, English, Mongolian, Thai, European language as between-subject factors) repeated-measures analyses of variance (ANOVA) to explore the main effect and interaction of the three variables.

## Results

**Figure 2:**
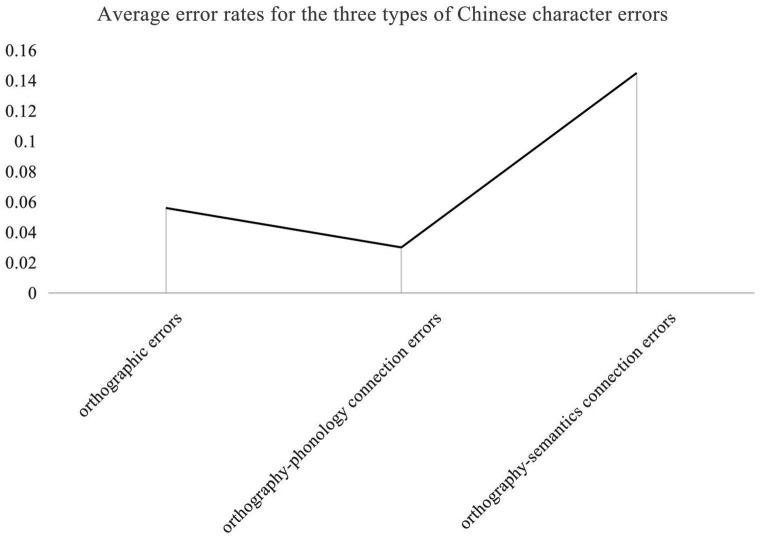
Average error rates for the three types of Chinese character errors.

The ANOVA revealed a main effect of Chinese character error types [*F*(2, 4,190) = 1101.52, *p* < 0.001, $\eta_{p}^{2}$ = 0.35] (presented in Figure 2), with higher average error rates of the orthography-semantics connection than that of orthography and the orthography-phonology connection, indicating that orthography, semantics, and phonology connected with each other in the process of CSL learners’ Chinese character output, and that orthography-semantics processing was the main obstacle for CSL learners’ processing of Chinese characters; a main effect of Chinese proficiency [*F*(2, 2095) = 37.40, *p* < 0.001, $\eta_{p}^{2}$ = 0.03], indicating that the processing of CSL learners’ Chinese characters was a gradual development process from low level to high level; and a main effect of language backgrounds [*F*(6, 2095) = 67.53, *p* < 0.001, $\eta_{p}^{2}$ = 0.16], the error rate was the highest for Japanese learners, followed by English and Korean learners. Russian, Mongolian, and European language learners had lower error rates, and Thai learners had the lowest error rates, indicating that there were significant differences in the output and processing of Chinese characters among CSL learners with different language backgrounds.

Multiple comparisons showed that the difference of Chinese character error rate of low level and high level learners was significant [MD = 0.05, *p* < 0.001], the difference of Chinese character error rate of middle level and high level learners was also significant [MD = 0.03, *p* < 0.001]. But the difference of Chinese character error rate of low level and middle level learners was not significant [MD = 0.02, *p* = 0.038]. Multiple comparisons revealed that there was significant difference of Chinese character error rate of Japanese and Korean, Thai, Russian, English, Mongolian, European language learners [MD = 0.11, *p* < 0.001, MD = 0.25, *p* < 0.001, MD = 0.14, *p* < 0.001, MD = 0.11, *p* < 0.001, MD = 0.15, *p* < 0.001, MD = 0.14, *p* < 0.001]. There was significant difference of Chinese character error rate of Thai and Korean, Russian, English, Mongolian, European language learners [MD = 0.14, *p* < 0.001, MD = 0.11, *p* < 0.001, MD = 0.14, *p* < 0.001, MD = 0.10, *p* < 0.001, MD = 0.11, *p* < 0.001]. However, the difference of Chinese character error rate of Korean and Russian, English, Mongolian, European language learners was not significant [MD = 0.03, *p* = 0.46, MD = 0.00, *p* = 1, MD = 0.04, *p* = 0.091, MD = 0.03, *p* = 0.394]. The difference of Chinese character error rate of Russian and English, Mongolian, European language learners was not significant [MD = 0.03, *p* = 0.11, MD = 0.01, *p* = 1, MD = 0.00, *p* = 1]. The difference of Chinese character error rate of English and Mongolian, European language learners was not significant [MD = 0.04, *p* = 0.01, MD = 0.03, *p* = 0.05]. The difference of Chinese character error rate of Mongolian and European language learners was also not significant [MD = 0.01, *p* = 0.998]. Multiple comparisons revealed that the difference of Chinese character error rate of orthography and orthography-phonology connection was significant [MD = 0.03, *p* < 0.001], the difference of Chinese character error rate of orthography and orthography-semantics connection was significant [MD = 0.09, *p* < 0.001], and the difference of Chinese character error rate of orthography-phonology connection and orthography-semantics connection was also significant [MD = 0.11, *p* < 0.001].

**Figure 3:**
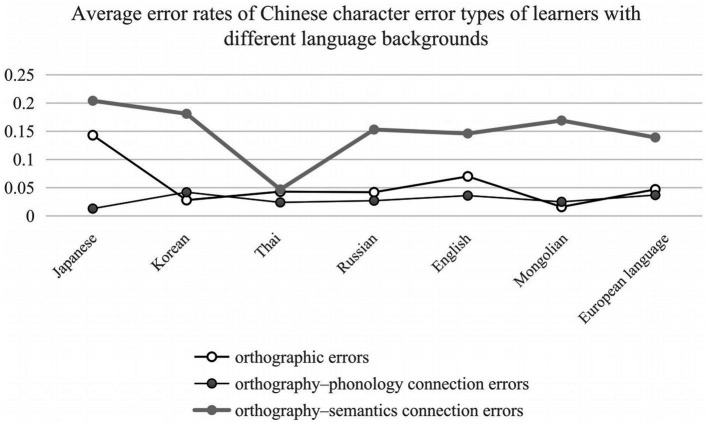
Average error rates of Chinese character error types of learners with different language backgrounds.

The ANOVA also revealed a significant interaction between Chinese character error types and Chinese proficiency [*F*(4, 4,190) = 23.64, *p* < 0.001, $\eta_{p}^{2}$ = 0.02], orthographic processing errors worked through the processing of different Chinese proficiency levels, phonological processing played a certain interference, and the role of orthographic processing was the largest; a significant interaction between Chinese character error types and language backgrounds [*F*(12, 4,190) = 47.86, *p* < 0.001, $\eta_{p}^{2}$ = 0.12] (shown in Figure 3), the error rate of orthography-semantics connection was higher than that of orthography and orthography-phonology connection among all learners of the seven language backgrounds. However, orthographic error rate was higher than that of the orthography-phonology connection for Japanese, Thai, Russian, English and European language learners. In contrast, the error rate of orthography-phonology connection was higher than that of orthography among Korean and Mongolian learners; and a significant interaction between Chinese proficiency and language backgrounds [*F*(12, 2,095) = 4.97, *p* < 0.001, $\eta_{p}^{2}\;$ = 0.03], as Chinese proficiency levels of Korean, Russian and Mongolian learners improved, the error rate decreased gradually. The error rate of Japanese, English and European language learners increased from low level to middle level, and then decreased. In contrast, the error rate of Thai learners decreased from low level to middle level, and then increased.

The simple effect analyses revealed that the error type effect of Chinese characters was all significant at three proficiency levels [*F*(2, 4,226) = 551.43, *p* < 0.001; *F*(2, 4,226) = 403.65, *p* < 0.001; *F*(2, 4,226) = 138.85, *p* < 0.001]. And the error type effect of Chinese characters was significant for Japanese, Korean, Thai, Russian, English, Mongolian and European language learners [*F*(2, 4,218) = 331.86, *p* < 0.001; *F*(2, 4,218) = 233.13, *p* < 0.001; *F*(2, 4,218) = 6.23, *p* = 0.002; *F*(2, 4,218) = 195.64, *p* < 0.001; *F*(2, 4,218) = 193.75, *p* < 0.001; *F*(2, 4,218) = 223.79, *p* < 0.001; *F*(2, 4,218) = 260.27, *p* < 0.001]. The simple effect analyses also revealed that language background effect was all significant at three proficiency levels [*F*(6, 2,097) = 33.04, *p* < 0.001; *F*(6, 2,097) = 24.67, *p* < 0.001; *F*(6, 2,097) = 14.43, *p* < 0.001].

The triple interaction between language background, Chinese proficiency, and Chinese character error types was also significant [*F*(24, 4,190) = 3.65, *p* < 0.001, $\eta_{p}^{2}$ = 0.02], indicating that CSL learners with different language backgrounds and Chinese proficiency levels showed significant differences in the processing of orthography, semantics, and phonology of Chinese characters. To further evaluate CSL learners’ error type effect, simple effect analyses were conducted. The error type effect of Chinese characters was significant at three proficiency levels for Japanese learners [*F*(2, 4,190) = 213.20, *p* < 0.001; *F*(2, 4,190) = 102.88, *p* < 0.001; *F*(2, 4,190) = 66.41, *p* < 0.001]. The error type effect of Chinese characters was significant at three proficiency levels for Korean learners [*F*(2, 4,190) = 95.07, *p* < 0.001; *F*(2, 4,190) = 100.24, *p* < 0.001; *F*(2, 4,190) = 51.42, *p* < 0.001]. The error type effect of Chinese characters was not significant at three proficiency levels for Thai learners [*F*(2, 4,190) = 3.23, *p* = 0.040; *F*(2, 4,190) = 1.93, *p* = 0.145; *F*(2, 4,190) = 1.72, *p* = 0.179]. The error type effect of Chinese characters was significant at three proficiency levels for Russian learners [*F*(2, 4,190) = 88.21, *p* < 0.001; *F*(2, 4,190) = 75.27, *p* < 0.001; *F*(2, 4,190) = 40.35, *p* < 0.001]. The error type effect of Chinese characters was significant at three proficiency levels for English learners [*F*(2, 4,190) = 106.78, *p* < 0.001; *F*(2, 4,190) = 103.09, *p* < 0.001; *F*(2, 4,190) = 23.79, *p* < 0.001]. The error type effect of Chinese characters was significant at three proficiency levels for Mongolian learners [*F*(2, 4,190) = 134.91, *p* < 0.001; *F*(2, 4,190) = 75.76, *p* < 0.001; *F*(2, 4,190) = 34.76, *p* < 0.001]. The error type effect of Chinese characters was significant at three proficiency levels for European language learners [*F*(2, 4,190) = 136.43, *p* < 0.001; *F*(2, 4,190) = 110.04, *p* < 0.001; *F*(2, 4,190) = 29.16, *p* < 0.001].

## Discussion

This study was designed to investigate how three variables of orthography, phonology and semantics affected Chinese characters production among 2,116 learners from 7 different L1s. First, this study found successful CSL learners’ Chinese character production was derived from connections between orthography, semantics, and phonology. Second, there was a significant contribution of semantics than orthography and phonology in the production of Chinese characters. Third, it was unexpected that connectionist models of languages rather than language distance affected production.

### Connections between orthography, semantics, and phonology

Chinese character production is an activity that produces the orthographic representation of characters according to phonological or semantic cues (Zhang and Roberts, 2019). This study did not deny the contribution of orthographic knowledge to character production but asserted that successful CSL learners’ Chinese character production derived from connections of orthography, semantics, and phonology, none of which were dispensable. The processing of production involves activating the units corresponding to an input pattern and letting activation pass to the output units *via* connections between orthography-phonology-semantics components (Seidenberg, 2005). Thus, the view of the connectionist model of character production holds that none of the three components is sufficient for skilled writing but rather that skill in all three components is necessary if writing skills are to advance. Only when the connection of various representations reached a particular strength could mutual activation and diffusion be realized, and the output of orthography could be realized efficiently (Deniz et al., 2019). This study found that the error rate of the orthography-semantics connection was much higher than that of the orthography and orthography-phonology connection. The connections of orthography and semantic radicals with semantic lexicon composed a complex connection system of orthography-semantics. These results are in line with the findings reported by Williams and Bever (2010), Tong and Yip (2015), and Wang et al. (2019) among native and CSL learners. The main reason may lie in the logographic nature of Chinese characters, which have systematic orthography-semantics mapping and opaque orthography-phonology correspondence. Consistent with the research by Zhang and Roberts (2019), phonological awareness did not directly contribute to the production of Chinese character. Moreover, language units are an orderly set of multiple semantic features, the connections of which are the core of the lexical knowledge system (Golestani et al., 2009; Xing, 2020). The perceptive process of language acquisition is from meaning to usage; however, the process of language production transfers from meaning to morphology, which depends on a clear connection between concepts and symbols and the selection of similar symbols. While orthographic knowledge was perceived as an effective strategy for learning new Chinese characters by CSL learners (Shen, 2005), the effective output of Chinese characters requires the construction of a rich sublexical network connection between orthographic representation and phonological representation, forming a complex sublexical system connected with the mental lexicon of orthography, phonology, and semantics in the cognitive process.

### Effects of semantics and phonology on the production of Chinese characters

While the inaccuracy of orthographic representation was associated with learners of different Chinese proficiency levels (Hamada and Koda, 2008; Lin and Collins, 2012), this is not the only difficulty in the production of Chinese characters; rather, orthography-semantic connection is the dominant factor in the processing of Chinese characters, suggesting evidence of “a semantic bias” (Williams and Bever, 2010). This study found that the number of analogy errors accounted for a large proportion of the orthography-semantic connection errors, with the number as high as 4,727 (see Supplementary Appendix Table 3), indicating that CSL learners tended to use similar or related Chinese characters to replace the target characters without effectively constructing connections between Chinese characters and concepts of mental lexicon. That is, the core relationship of the language system of the connection network between symbols and concepts is imperfect. While the number of errors in sublexical problems of semantic radicals was 196, accounting for only 3.98%. Chinese has a large number of homophones, words with the same sound but different meanings, and the prevalence of homophones in Chinese adds to the complexity of character acquisition (Kuo et al., 2015). This study found that orthography-phonology connection was also an obstacle for CSL learners in Chinese character production (McBride-Chang et al., 2004). Among the orthography-phonology connection errors, homophone substitution errors and phonetic radical errors were 790 and 253, covering 75.74 and 24.26%, respectively. Phonological representation and orthographic representation cannot be automatically activated; thus, CSL learners produce homophonic or near phonological characters. In addition, the error rate of the orthography-phonology connection was higher than that of orthography among Korean and Mongolian learners. First, the reason might lie in the fact that CSL learners have a small vocabulary and do not establish the corresponding entries in the mental lexicon, making the activation intensity of the orthographic representation of the target character lower than that of homophones and at a disadvantage in competition with homophones, resulting in the incorrect output of homophones in the production process (Wang et al., 2012). Second, this may be related to the lexical representation and processing characteristics of CSL learners. To summarize, semantics and phonology play different roles in the production of Chinese characters.

### Effects of language background on the production of Chinese characters

Previous studies on teaching Chinese characters as a second language have divided learners into the Sinosphere and non-Sinosphere (Hsiao et al., 2015). It is generally believed that CSL learners in the Sinosphere, who are influenced by traditional Chinese cultural beliefs characterized by Confucianist social and moral ethics or Taoist or Mahayana Buddhist religious beliefs, as embodied in the text using Chinese characters (Hsiao et al., 2015), perform better than learners in the non-Sinosphere (Lin and Collins, 2012; Tong et al., 2016; Zhang and Roberts, 2021a,b). A similar view of language distance was clearly stated by Koda (1996), who noted that “Ll-L2 orthographic distance not only influences overall performance differences among learners from related and unrelated L1 orthographic backgrounds but also underscores the ways in which L1 orthographic knowledge facilitates L2 word recognition” (p. 458). Contrary to previous studies, this study found that CSL learners with different language backgrounds showed significant differences in the production of Chinese characters. Specifically, the error rate of CSL learners in the Sinosphere, represented by Japanese learners, was the highest. Indeed, Japan uses a set of modified Chinese characters for the “Kanji” script that is supplemented by syllabaries scripts of Hiragana and Katakana. However, their understanding of the motivations of character formation and the connections between orthography, semantics, and phonology had no essential difference compared with learners in the non-Sinosphere (Wydell et al., 1993; Chen, 2001; Wan, 2003; Rose, 2013; Sakurai et al., 2021). They paid more attention to the correspondence between phonology and semantics than the connections between orthography and semantics (Chen, 2001). The main reason may lie in the fact that Chinese characters are used as syllabic characters in Japan, which is essentially different from the syllable-morpheme characters in written Chinese (Sakurai et al., 2021). Moreover, Japanese learners tend to view Chinese compound words as a whole instead of dividing the words into single Chinese characters; thus, they lack a real understanding of the words that prevents them from correctly writing the characters that make up the whole words. Even within the Sinosphere, learners perform differently in the productive process. Japanese learners have the highest error rate of the orthography-semantics connection, followed by orthographic errors and orthography-phonology connection errors, while Korean learners have the highest error rate of the orthography-phonology connection, followed by orthographic errors, and the lowest error rate of the orthography-semantics connection (Quan, 2006). These results suggest that the sublexical network connections between orthographic representation and semantic representation have not been constructed among Japanese learners, yet Korean learners cannot automatically activate orthographic representation and phonological representation.

In the non-Sinosphere, while there is no big difference in the average error rate among English, Russian, Mongolian, and European language learners, there are differences in the types of Chinese character errors. Although the error rate of orthography-semantics connection is the highest, the error rate for the orthography-phonology connection of Mongolian learners is higher than that of orthography. Nonetheless, the orthographic error rate of English, Russian and European language learners is higher than that of the orthography-phonology connection. This indicates that Mongolian learners have significant problems in the connection and processing of phonological lexicons, yet orthographic representation and processing are the main obstacles to the production of Chinese characters for English, Russian and European language learners. We are also interested in Thai learners who have the lowest average error rate. The problems encountered in learning Chinese characters are different among learners from different countries and cultures, resulting in differences in the output of Chinese characters. This awaits further investigation. To summarize, the results of this study indicate that orthographic knowledge and language distance (Hamada and Koda, 2008) are not the main factors affecting Chinese character production, whereas different connection models of orthography, semantics, and phonology of languages are the key points. Future research on Chinese character teaching and learning should break through the boundary between the Sinosphere and non-Sinosphere to further explore the representation mechanism of Chinese characters.

## Conclusion

In conclusion, our findings reveal the effects of orthographic, phonological, and semantic processing in the production of Chinese characters among CSL learners. The current study emphasizes that successful Chinese character production derives from the connections of orthography, semantics, and phonology, of which semantics plays the most significant role. The results also point to different connection models of languages rather than first language orthographic experience on second language decoding. These new findings enrich our understanding of the acquisition of literacy skills for CSL learners.

## Data availability statement

The original contributions presented in the study are included in the article/Supplementary material, further inquiries can be directed to the corresponding author.

## Ethics statement

The studies involving human participants were reviewed and approved by the Institute on Educational Policy and Evaluation of International Students, Beijing Language and Culture University. The ethics committee waived the requirement of written informed consent for participation.

## Author contributions

LZ performed the statistical analysis and wrote the manuscript. HX provided advice and revised the manuscript critically. All authors contributed to the article and approved the submitted version.

## Funding

This project was supported by the Humanities and Social Sciences Research Planning Project by the Ministry of Education (No. 20YJAZH110).

## Conflict of interest

The authors declare that the research was conducted in the absence of any commercial or financial relationships that could be construed as a potential conflict of interest.

## Publisher’s note

All claims expressed in this article are solely those of the authors and do not necessarily represent those of their affiliated organizations, or those of the publisher, the editors and the reviewers. Any product that may be evaluated in this article, or claim that may be made by its manufacturer, is not guaranteed or endorsed by the publisher.
