# Supplementary material for: The interaction of orthography, phonology and semantics in the process of second language learners’ Chinese character production
Source: Front Psychol. 2023 Mar 2;14:1076810. doi: 10.3389/fpsyg.2023.1076810 (PMC10017467; doi:10.3389/fpsyg.2023.1076810)
Supplement: Supplementary file 1 [file Table_1.docx]

**TABLE 1** | Target Chinese characters.

| **Target Chinese characters** | | | | | | | | | | | | | | | |
| --- | --- | --- | --- | --- | --- | --- | --- | --- | --- | --- | --- | --- | --- | --- | --- |
| 许 | 毕 | 团 | 努 | 成 | 尽 | 善 | 联 | 脑 | 诉 | 息 | 收 | 礼 | 影 | 近 | 乐 |
| 特 | 熟 | 平 | 专 | 内 | 现 | 量 | 许 | 绍 | 任 | 虽 | 论 | 提 | 相 | 展 | 情 |
| 决 | 基 | 交 | 神 | 处 | 申 | 材 | 办 | 园 | 备 | 真 | 轻 | 战 | 注 | 迎 | 记 |
| 复 | 积 | 造 | 考 | 最 | 关 | 通 | 组 | 谈 | 术 | 境 | 待 | 务 | 活 | 照 | 康 |
| 快 | 命 | 远 | 温 | 幸 | 慰 | 受 | 健 | 况 | 加 | 费 | 席 | 直 | 培 | 支 | 批 |
| 静 | 首 | 握 | 气 | 努 | 暖 | 实 | 交 | 外 | 烦 | 岗 | 基 | 岁 | 态 | 片 | 报 |
| 必 | 务 | 社 | 负 | 现 | 论 | 支 | 解 | 愉 | 顺 | 外 | 受 | 识 | 规 | 书 | 保 |
