# Supplementary material for: The interaction of orthography, phonology and semantics in the process of second language learners’ Chinese character production
Source: Front Psychol. 2023 Mar 2;14:1076810. doi: 10.3389/fpsyg.2023.1076810 (PMC10017467; doi:10.3389/fpsyg.2023.1076810)
Supplement: Supplementary file 2 [file Table_2.docx]

**TABLE 2** | Distribution of sampling data.

| **Language backgrounds** | **Chinese proficiency** | | | **Total** |
| --- | --- | --- | --- | --- |
|  | Low level | Middle level | High level |  |
| Japanese | 79 | 78 | 73 | 230 |
| Korean | 72 | 72 | 70 | 214 |
| Russian | 110 | 103 | 60 | 273 |
| English | 160 | 127 | 118 | 405 |
| Mongolian | 67 | 79 | 54 | 200 |
| Thai | 80 | 105 | 70 | 255 |
| European language | 224 | 211 | 104 | 539 |
| **Total** | 792 | 775 | 549 | 2116 |
