# Supplementary material for: The interaction of orthography, phonology and semantics in the process of second language learners’ Chinese character production
Source: Front Psychol. 2023 Mar 2;14:1076810. doi: 10.3389/fpsyg.2023.1076810 (PMC10017467; doi:10.3389/fpsyg.2023.1076810)
Supplement: Supplementary file 3 [file Table_3.docx]

**TABLE 3** | Numbers and proportions of errors.

| **Orthographic errors** | | | **Orthography-phonology connection errors** | | | **Orthography-semantics connection errors** | | |
| --- | --- | --- | --- | --- | --- | --- | --- | --- |
| Error category | Numbers | Proportions | Error category | Numbers | Proportions | Error category | Numbers | Proportions |
| Structural errors | 137 | 7.24% | Homophone substitution errors | 790 | 75.74% | Analogy errors | 4727 | 96.02% |
| Component errors | 664 | 35.10% | Sublexical errors of  phonetic radicals | 253 | 24.26% | Sublexical errors of semantic radicals | 196 | 3.98% |
| Stroke errors | 1091 | 57.66% | **Total** | 1043 |  | **Total** | 4923 |  |
| **Total** | 1892 |  |  |  |  |  |  |  |
